# Subjective assessment of sleep quality in adult patients with hereditary angioedema

**DOI:** 10.3389/fneur.2025.1555562

**Published:** 2025-04-16

**Authors:** Esra Karabiber, Ezgi Yalçın Güngören, Ahmet Özen, Safa Barış, Elif Karakoc-Aydiner

**Affiliations:** ^1^Department of Chest Disease, Division of Adult Allergy and Immunology, Pendik Training and Research Hospital, Istanbul, Türkiye; ^2^Division of Pediatric Allergy and Immunology, Department of Pediatrics, School of Medicine, Marmara University, Istanbul, Türkiye; ^3^The Istanbul Jeffrey Modell Diagnostic Center for Primary Immunodeficiency Diseases, Istanbul, Türkiye; ^4^The Isil Berat Barlan Center for Translational Medicine, Istanbul, Türkiye

**Keywords:** hereditary angioedema, insomnia, sleep quality, sleep disorders, sleep disturbance

## Abstract

**Background:**

Hereditary angioedema (HAE) is a rare disorder characterized by recurrent swelling episodes, including painful abdominal attacks and life-threatening angioedema of the larynx that significantly affects patients’ quality of life, including sleep. Sleep disorders have not yet been elucidated in HAE patients.

**Methods:**

This study evaluated sleep quality and insomnia by comparing attack-free periods with abdominal, head-neck, and extremity attacks. Sleep quality and insomnia were assessed using two validated questionnaires with the Basic Scale on Insomnia and Quality of Sleep (BaSIQS) and the Pittsburgh Sleep Quality Index (PSQI): sleep latency, difficulty falling asleep, night awakenings, problems returning to sleep, and overall sleep quality.

**Results:**

The study included 23 HAE patients; the median age was 31 years [interquartile range (IQR): 25–37], with a female predominance (*n* = 16, 69.5%). During the attack-free period, the median PSQI total score was 5 (IQR: 3.75 7.25), with 56.2% of the participants (*n* = 13) classified as good sleepers. Total PSQI scores significantly increased during attack periods compared to the attack-free period (median: 10 for abdomen, 8.5 for extremities, and 7.5 for head-neck; *p* < 0.001 for all). Whereas during the attack-free period, only 40.9% of patients exhibited good sleep quality (BaSIQS <9), with a median score of 10 (IQR: 7–14.2).Scores of BaSIQS significantly increased during angioedema attacks, with medians of 15 (extremity), 16 (abdominal), and 17 (head-neck), reflecting a notable decline in sleep quality. Among the components of PSQI, compared to the attack-free period, scores except the one assessing the need for medication to sleep all domains showed statistically significant increase.

**Conclusion:**

This study demonstrates that poor sleep quality, prolonged sleep latency, and increased awakenings are prevalent among HAE patients. Screening for sleep disorders and targeted interventions may help improve disease control and overall quality of life in HAE patients.

## Introduction

Hereditary angioedema (HAE) is a rare genetic disorder characterized by recurrent episodic angioedema of the cutaneous and subcutaneous tissue in various organs. Although the swelling is self-limited and resolves spontaneously in 3–5 days, laryngeal involvement may cause fatal asphyxiation (1, 2). Pathogenic variants in the *SERPING1* gene result in either a reduced synthesis of C1 esterase inhibitor protein (HAE type I), the most common type, or a dysfunctional C1 inhibitor protein (HAE type II). Additionally, the newly classified type of HAE is characterized by normal levels and function of the C1 inhibitor protein.

Due to a lack of awareness, diagnosis of HAE is frequently delayed, potentially leading to substantial psychological distress and fear of subsequent attacks. The disease following the diagnosis of HAE still imposes a considerable psychosocial burden, characterized by increased disease-related stress, depression, anxiety, and a notably diminished health-related quality of life (HRQoL) (3). In a Canadian study on HAE, psychosocial burden and HRQoL scores were poorly affected compared to the general population, whereas sleep was assessed briefly and reported to be impaired in every three out of four participants (4). This is probably related to intensely painful, unpredictable attacks often necessitating emergency medical care, even in case long-term or short-term prophylactic measures are undertaken. In addition to depression, anxiety, and impaired HRQoL, chronic diseases such as rheumatic, autoimmune, lung, cardiovascular, and primary immunodeficiencies have been linked to sleep disorders (5–9). Up to the current date, there is only one study evaluating sleep disorders among non-severe HAE patients and reported a higher incidence of sleep disorders. The most prevalent conditions were intermediate and high-risk obstructive sleep apnea (OSA) and insomnia in participants (10). These preliminary results underscore the prevalence of sleep disorders and poor sleep quality among HAE patients, suggesting that further evaluation of potential sleep disturbances should be integrated into the clinical management of these patients.

To our knowledge, the prevalence of subjective sleep quality in HAE patients has not yet been systematically assessed. This study addresses this gap by examining sleep quality and disturbances in adult HAE patients.

## Methods

This prospective study assessed subjective sleep quality and sleep characteristics in adult patients diagnosed HAE with C1 inhibitor deficiency or dysfunctional (HAE type I, II) and normal C1 inhibitor from a single center at a tertiary hospital in Istanbul between December 2023 and May 2024. The inclusion criteria were the following: (i) ≥18 years old, (ii) diagnosis of HAE (I–II and normal C1 inhibitor), and (iii) being literate. The results of the C1 inhibitor level, function, and diagnosis of patients were given in Supplementary Table S1. None of the HAE patients already had a physician-diagnosed sleep disorder and were on sleep medication prescriptions. Any HAE patients with additional chronic diseases were excluded from the study.

The diagnosis and management of HAE was made according to the national and global guidelines (11, 12). Clinical and demographic data was collected from medical records, including frequency of attacks, attack region, family history, laboratory features, treatment regimens, short and long-term prophylaxis, and attack treatment. Disease activity was evaluated using the Angioedema Activity Score (AAS) and the Angioedema Control Test (AECT) based on their condition over the past 4 weeks (13, 14). Sleep quality was assessed using two validated questionnaires: the Basic Scale on Insomnia and Quality of Sleep (BaSIQS) and the Pittsburgh Sleep Quality Index (PSQI) (15–18). Patients completed the PSQI and BaSIQS questionnaires during attack-free periods and during experiencing attacks for each region, including the extremities, abdomen, and head-neck.

The local Institutional Review Board approved our study protocol under protocol number 1519, dated 09.2023. Written informed consent was obtained from all patients before initiating any study procedure. Each patient provided written informed consent, and all studies were conducted per the principles of the Declaration of Helsinki.

The AECT comprises four questions that assess the frequency of symptoms, the impact on quality of life, the unpredictability of attacks, and the level of control achieved with current treatment. Each question is rated from 0 to 4, yielding a total possible score ranging from 0 (no control) to 16 (complete control). A score of 10 has been identified as the threshold for distinguishing between poorly controlled and well-controlled conditions (13, 14, 19). The AAS items rated from 0 to 3, accumulating a 28-day total score from 0 to 420, where a higher score reflects increased disease activity (13, 14).

The BaSIQS, initially developed in Portuguese, encompasses seven items that evaluate sleep onset and maintenance difficulties and a subjective assessment of sleep quality and depth over the preceding month. Each item is scored using a 5-point Likert scale ranging from 0 to 4, except for the final two items, scored in reverse. The total score, derived by summing the item scores, varies from 0 to 28, where higher scores indicate worse sleep quality. A threshold score above 9 discriminates the cut-off value between good and poor sleep quality. The PSQI is a self-administered questionnaire designed to assess sleep quality and disturbances during the prior month. It comprises 24 items, of which 19 are self-reported, and five are completed by a roommate. The questionnaire generates a total score and seven component scores reflecting subjective sleep quality, sleep latency, sleep duration, habitual sleep efficiency, sleep disturbances, use of sleep medication, and daytime dysfunction. Each component is rated on a scale from 0 to 3, with the total score ranging from 0 to 21; higher scores signify more severe disturbances. A global score exceeding 5 is described as the cut-off value to differentiate between good and poor sleepers.

### Statistical analysis

Data analyses were performed using SSPS statistical software (IBM SPSS Statistics for Windows, Version 22.0. Armonk, NY: IBM Corp.) and GraphPad Prism 8 (GraphPad Software Inc., San Diego, California, United States). A homogeneity test was performed using the Shapiro–Wilk test. Continuous variables were analyzed using Mann–Whitney *U* and Kruskal–Wallis tests. Categorical variables were analyzed using the chi-square or Fisher exact test. Spearman correlation test was used to analyze correlation. Wilcoxon and Kruskal–Wallis tests were used to compare dependent variables. The differences were considered statistically significant at *p* < 0.05.

## Results

A total of 23 HAE adult patients were enrolled in the study. The median age of the participants was 31 years [interquartile range (IQR): 25–37], with a female predominance (*n* = 16, 69.5%). The characteristics of the HAE patients are summarised in Table 1. The majority of cases were diagnosed as HAE type I (*n* = 19, 82.6%), while normal C1 inhibitor HAE was confirmed in only one subject. The median diagnostic delay for HAE was 12 years, ranging from 5 to 19 years. Icatibant was prescribed as an on-demand treatment to all participants, and three participants were exceptional due to pregnancy and breastfeeding. Abdominal attacks were the most frequently reported initial symptom in HAE patients (*n* = 12, 52.1%). Laryngeal attacks were reported in 15 patients, accounting for 70% of the cases throughout their lives. Despite the ambulatory subcutaneous acute attack treatment with icatibant, the median annual frequency of hospital admissions for HAE attacks was five. The average time for complete recovery of acute attacks was 42 h with icatibant treatment. The annual icatibant need among the participants was 48 doses at the median, ranging from 24 to 96. The median score for the AECT among the HAE patients was 8 (IQR: 4.5–9.75). Of the 20 patients, only 5 (25%) had well-controlled disease. The median AAS was 49.5 (IQR: 17–96), with a maximum score of 200.

**Table 1:** Demographics and clinical features of HAE patients (*n* = 23).

Characteristics	Median (IQR)
Current age (years)	31 (25–37)
Sex^a^ [female (%)]	16 (69.5)
Age at symptom onset (years)	8 (4–14)
Age at diagnosis (years)	22 (14–26)
Delay in diagnosis (years)	12 (5–19)
Type of HAE^a^ *n* (%)	23
Type I	19 (82.6)
Type II	3 (13)
Normal C1 inhibitor	1 (4.3)
Attacks frequency (*n*)	
Extremities/year	36 (24–72)
Abdomen/year	24 (6–38)
Head-neck/year	2 (1–3)
Annual admission to the hospital (*n*)	5 (0–7)
Recovery of attacks with icatibant (hours)	48 (24–48)
Long term prophylaxis (*n*)	
Danazol	2 (8.6)
C1 inhibitor concentrate	2 (8.6)
Attack treatment, *n* (%)	
Icatibant	21 (91.3)
Icatibant + C1 inhibitor concentrate	1 (4.3)
Annual icatibant administration (*n*)	48 (8–96)
AECT scores (*n* = 22)	8 (4.5–9.75)
AAS scores (*n* = 22)	49.5 (17–96)
BaSIQS scores	
Without attacks (*n* = 22)	10 (7–14.5)
With attacks (*n* = 59)	16 (14–19)
Extremities (*n* = 20)	15 (10–19)
Abdomen attacks (*n* = 22)	16 (14–21)
Head-neck (*n* = 17)	17 (14–21)
PSQI scores	
Without attacks (*n* = 22)	5 (3.75–7.25)
With attacks (*n* = 59)	9 (7.5–12)
Extremities (*n* = 20)	8.5 (6–10)
Abdomen attacks (*n* = 22)	10 (8–12.5)
Head-neck (*n* = 17)	10 (7.5–14)

HAE, hereditary angioedema; AECT, Angioedema Control Test; AAS, Angioedema Activity Score; PSQI, Pittsburg Sleep Quality Index; BaSIQS, Basic Scale on Insomnia and Quality of Sleep; IQR, interquartile range, No., number.

a Data indicated as frequency in number and percentage.

**Figure 1:**
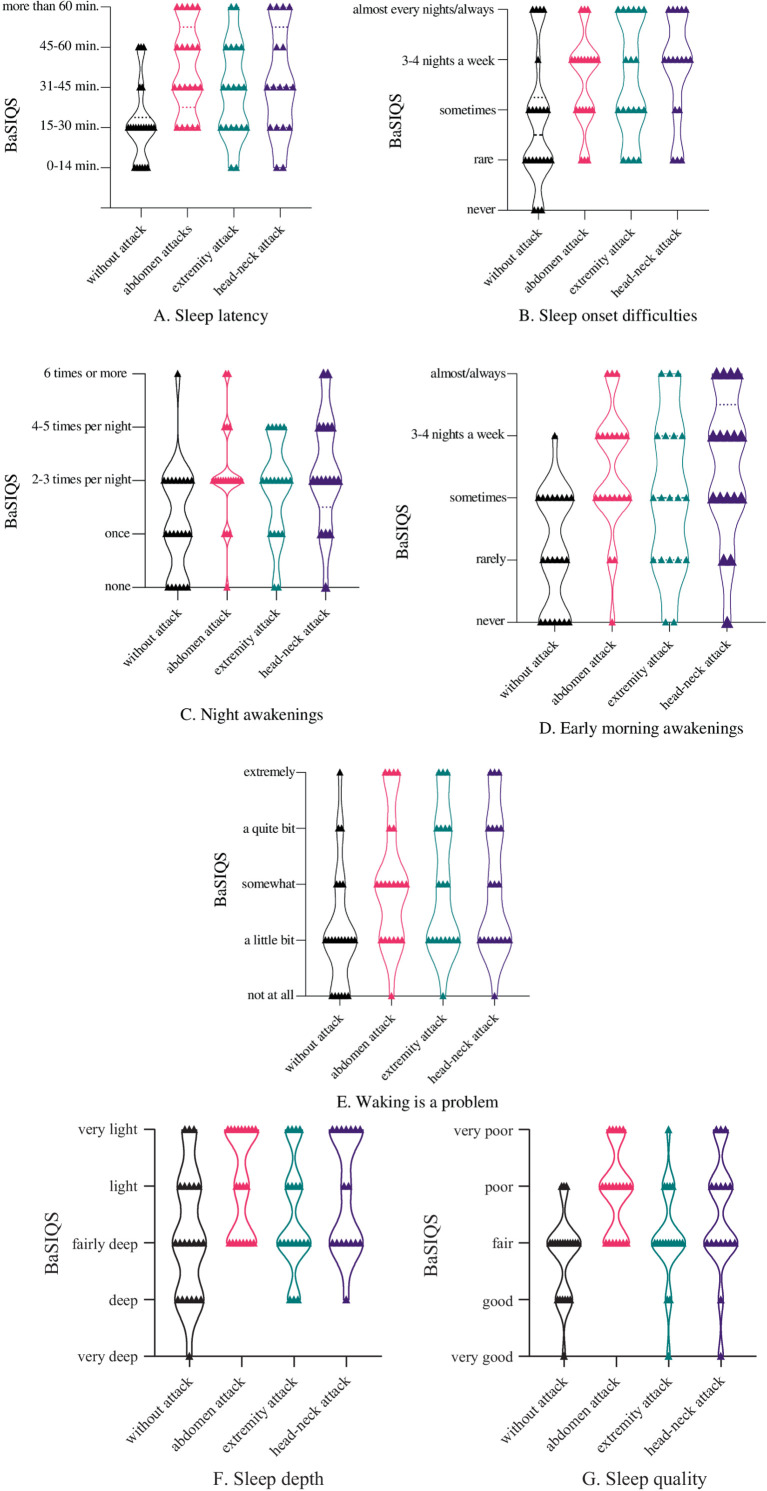
Basic Scale on Insomnia Complaints and Quality of Sleep (BaSIQS) in HAE patients in attack-free period and angioedema attacks in extremities, abdomen, and head-neck, which shows patients’ answers based on seven subcomponents: **(A)** sleep latency, **(B)** sleep onset difficulties, **(C)** night awakenings, **(D)** early morning awakenings, **(E)** waking is a problem, **(F)** sleep depth, **(G)** sleep quality. Categorical variables were analyzed using the Fisher–Freeman–Halton’s exact test. The differences were considered statistically significant at *p* < 0.05. The figure depicts values as median with range.

According to the BaSIQS, nine patients (40.9%) scored below nine during the attack-free period, indicating good sleep quality. During the attack-free period, only five patients (22.7%) reported taking over 30 min to fall asleep, with all falling asleep within an hour (Figure 1A). The percentage of patients taking over an hour to fall asleep was 23.8% (*n* = 5) during abdominal attacks, 23.5% (*n* = 4) during head-neck attacks, and 10.5% (*n* = 2) during extremity attacks (Figure 1A). Moreover, significantly more patients experienced prolonged sleep latency during extremity attacks compared to attack-free periods (*p* = 0.002). However, sleep onset was significantly delayed during attacks, affecting 76.2% (*n* = 17) of patients during abdominal attacks, 64.7% (*n* = 11), and 57.9% (*n* = 11) during head-neck attacks and extremity attacks. Concerning sleep onset difficulties, only three patients reported “never” even during attack-free periods, whereas no patients selected the “never” option during any attack type (abdominal pain, head-neck, or extremity) (Figure 1B). In terms of night awakenings, seven patients (31.8%) indicated “none” during attack-free periods, on the contrary, only one patient (4.8 and 5.9%, respectively) for each of abdominal and head-neck attacks (Figure 1C). For early morning awakenings, six patients reported “never” during attack-free periods (Figure 1D). Regarding wakening (early/night) as a problem, six patients indicated “not at all” during attack-free periods, while only one for each patient reported “not at all” during both abdominal and extremity attacks (Figure 1E). Seven patients (31.8%) rated their sleep quality as “good or very good” during attack-free periods. However, no patients selected these options during abdominal attacks. Only two and three patients, respectively, rated their sleep quality as “good or very good” during head-neck and extremity attacks (Figure 1F). In terms of sleep depth, seven patients (31.8%) reported “deep or very deep” sleep during attack-free periods, while only one patient reported “deep” sleep during abdominal attacks (Figure 1G).

**Figure 2:**
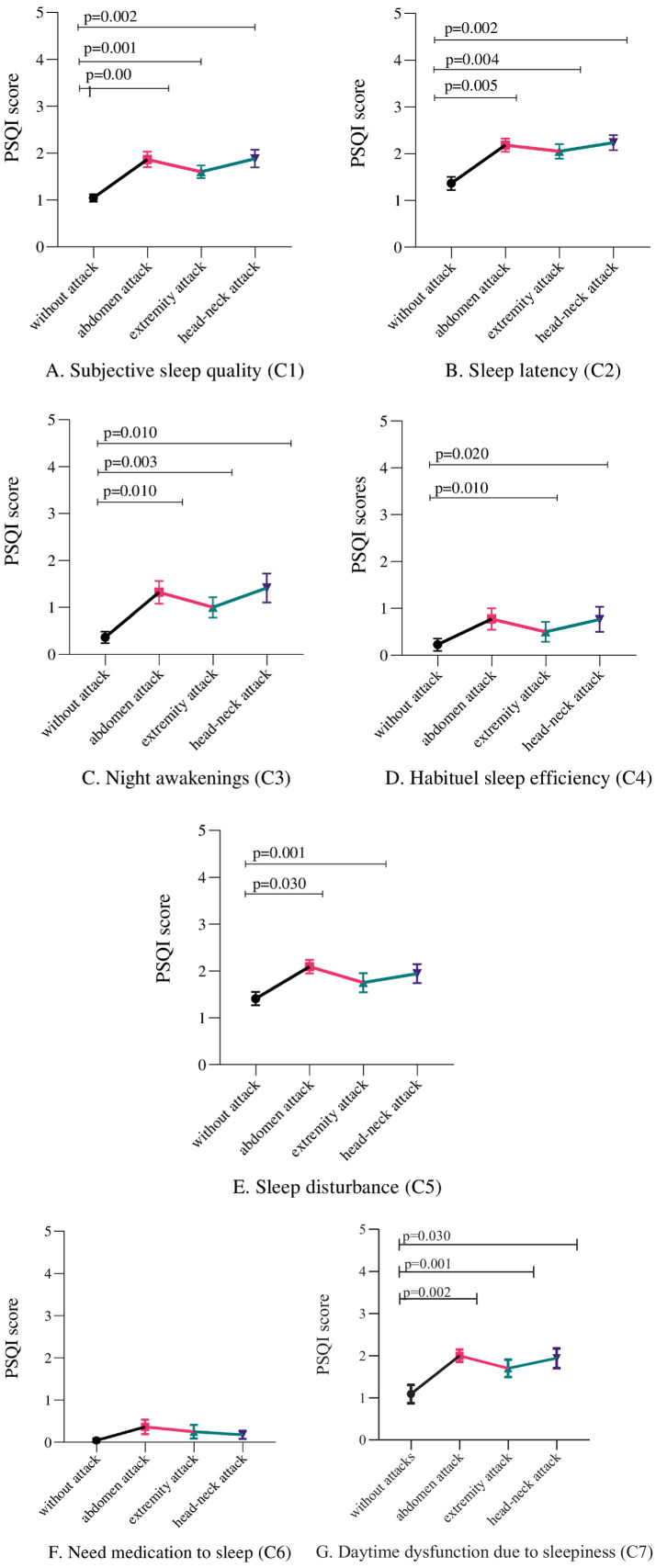
Pittsburg Sleep Quality Index (PSQI) scores during attacks on the abdomen, extremities, and head-neck compared to attack-free periods for seven components (C1–C7): **(A)** subjective sleep quality, **(B)** sleep latency, **(C)** sleep duration, **(D)** habitual sleep efficiency, **(E)** sleep disturbances, **(F)** use of sleep medication, and **(G)** daytime dysfunction. Variables were analyzed using Wilcoxon signed-rank tests. The differences were considered statistically significant at *p* < 0.05. The figure depicts values as mean ± standard error.

According to PSQI questionnaires, during the attack-free period, 13 patients’ total scores indicated them as good sleepers, accounting for 56.2% of the group, whereas the median PSQI score was 5 (IQR; 3.75–7.25) in attack-free HAE patients (Table 1). When evaluating the PSQI components, two individuals rated their sleep quality as “very bad” during the attack-free period, while 11 individuals during extremity and head-neck attack and 16 during an abdominal attack rated their sleep quality as either “fairly bad or very bad.” Subjective sleep quality significantly declined during angioedema attacks, with the greatest impairment observed in abdominal attacks (Figure 2A). Among the components of the PSQI, all scores except for the one assessing the need for medication to sleep showed a statistically significant increase during abdominal attacks compared to the attack-free period (Figures 2B–G). In assessing sleep duration, it was found that one individual during the attack-free period, 7, 10, and 8 individuals during an extremity attack, an abdominal attack, and a head-neck attack reported sleeping for 6 h or less (Figures 2B–G). When comparing the components across different attack types, such as abdominal, extremity, and head-neck attacks, the type of attack did not significantly influence the PSQI components.

**Figure 3:**
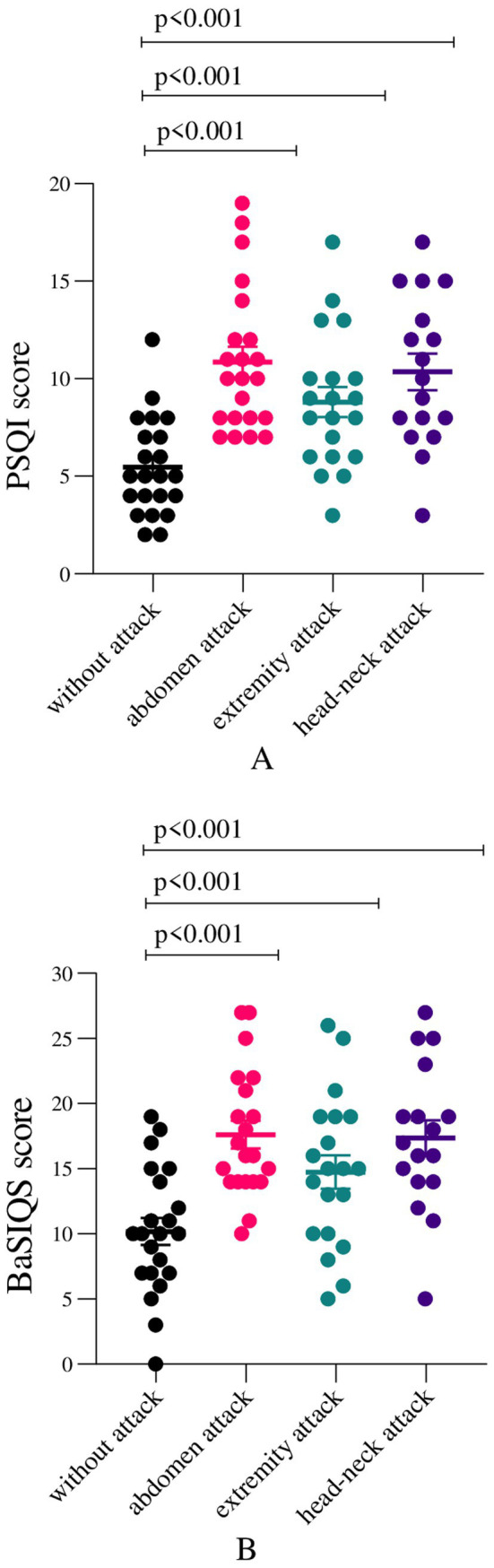
**(A)** Total Pittsburg Sleep Quality Index (PSQI) and **(B)** Basic Scale on Insomnia Complaints and Quality of Sleep (BaSIQS) scores among HAE patients with various attack types versus attack-free. Variables were analyzed using Wilcoxon signed-rank tests. The differences were considered statistically significant at *p* < 0.05. The figure depicts values as mean ± standard deviation.

The comparison of total PQSI scores and BaSIQS scores during attack-free periods and any angioedema attacks is shown in Figures 3A,B and Table 1. According to both questionnaires, total sleep scores were statistically superior to those related to extremity, abdominal, and head-neck attacks compared to attack-free periods. Comparisons of total PSQI scores between the attack-free period and various attack periods showed significant increases (median scores of 5 vs. 10 for abdomen, 8.5 extremities, and 7.5 for head-neck), highlighting a marked rise in total PSQI scores (*p* < 0.001 for all comparisons) (Figure 3A). However, comparing total PSQI scores across different types of attacks, including abdomen, extremities, and head-neck, did not reveal any significant differences. Furthermore, a significant correlation was found between BaSIQS scores and gender, with females showing higher scores (median 10, IQR: 7–15) compared to males (median 8, IQR: 3–10) (*p* = 0.018). Additionally, when comparing attack types, the BaSIQS scores during abdominal attacks were significantly higher than the ones in extremity attacks (*p* = 0.014). No significant correlations were identified between BaSIQS and PSQI scores concerning age, number of attacks, duration of icatibant effect, annual hospital visits, AECT, or AAS.

## Discussion

This study investigated sleep quality, sleep disturbance, and insomnia in adult patients with HAE. To our knowledge, this is the first study about patient-reported subjective sleep quality in a rare disease group of HAE. According to our study’s findings, almost half of HAE patients had poor sleep quality and insomnia in attack-free periods. Moreover, the total PQSI and the BaSIQS scores worsened during the extremity, abdomen, and head-neck attacks. Among the components of PSQI, scores except the one assessing the need for medication to sleep showed an increase compared to the attack-free period. Moreover, BaSIQS and PSQI scores did not correlate to other factors affecting sleep, such as age, number of attacks, duration of icatibant effect, annual hospital visits, AECT, or AAS.

The diagnostic delay observed in our study is consistent with previously reported findings from other countries, highlighting a significant delay between symptom onset and diagnosis in HAE patients (20, 21). This delay underscores the need for improved awareness and earlier disease recognition to facilitate timely intervention. In our study, only 25% of patients had well-controlled diseases. This result indicates that patients experienced frequent attacks during the study period and highlights the challenges in achieving optimal disease management. Despite this, there was no correlation between disease control scores (AECT and AAS) and sleep quality, suggesting that factors other than disease severity may play a role in sleep disturbances. In one study, similarly, most sleep disorders were observed even in the non-severe HAE group, and sleep quality did not differ statistically between patients with severe and non-severe disease (10).

Sleep disorders are prevalent among adults and are associated with detrimental outcomes, including diminished quality of life and increased risk of motor vehicle accidents. Current guidelines suggest an optimal sleep duration of 7 to 9 h for adults, yet approximately 40% of Americans report sleeping 6 h or less each night (22). Insomnia, the most prevalent sleep disorder, affects about 33% of adults with symptoms, while 6 to 10% fulfill the criteria for a diagnosis of insomnia disorder. Chronic insomnia is defined by difficulties initiating sleep (initiation time exceeding 30 min), maintaining sleep, or early morning awakenings with associated daytime consequences, occurring at least three times per week for a minimum of 3 months. According to a Turkish national survey, the insomnia rate was 15.3% in adults and more affecting women (17.6%) compared to men (23). The current study demonstrated that poor sleep quality, prolonged sleep latency, and increased awakenings are prevalent among HAE patients, even in attack-free periods. Polysomnography is the definitive test for objectively assessing sleep quality. However, it is expensive and takes time to evaluate. Questionnaires, such as the Pittsburg Sleep Quality Index and the Basic Scale on Insomnia and Quality of Sleep, were developed to assess and screen subjective sleep quality and insomnia. Both of these validated questionnaires designed specifically for sleep domains were used to evaluate sleep in our HAE cohort.

Sleep and sleep quality may be affected by various factors, including anxiety, depression, age, sex, and angioedema attacks. Studies assessing depression at HAE reported a significant increase in the prevalence of sleep disorders as a domain along with depression among females, with an observed rate of 40% (24). Additionally, some other studies indicate the prevalence of depression, ranging from 14 to 42.5%, in patients with HAE (25, 26). A Canadian study reported a 76.5% rate of sleep disturbances in HAE patients, potentially linked to the nocturnal onset of attacks or as a consequence of psychological distress. Anxiety is notably prevalent among those experiencing severe or frequent HAE attacks and may significantly impair their quality of life (26, 27).

A recent study in Japan found that HAE attacks most commonly occurred between 6 and 7 a.m., likely due to the attacks commencing during sleep and being recognized upon awakening. The study also highlighted sleep deprivation as a potential trigger for these episodes (28). In the same study, the angioedema quality of life score was 37.1 ± 22.9, indicative of moderate to severe impairment. Additionally, out of 58 recorded attacks, 10 occurred between midnight and 5 a.m. (28). Furthermore, sleep deprivation was reported as a trigger for episodes in the study. In our study, all patients stated awakening during the night due to episodes of angioedema. Sleep disturbances may play a significant role in the increased attack frequency and the challenges in achieving disease control. This interplay may contribute to a vicious cycle of sleep deprivation and recurrent attacks, further exacerbating disease burden and impairing overall quality of life.

In a study of 63 HAE patients, abdominal attacks were associated with substantial pain, the most severe sleep disturbances, likely due to the intense pain and gastrointestinal symptoms accompanying these episodes. All HAE attacks were associated with substantial pain, averaging 8.4 on a subjective scale from 1 (minimal) to 10 (maximal). Three-quarters of these episodes included symptoms of nausea, vomiting, and abdominal distension, and 41% involved diarrhea. The attacks typically lasted 4 days, peaking on the second day, and began with prodromal symptoms before fully resolving (29). The relationship between pain and sleep quality has been documented across various chronic conditions, including rheumatic diseases (30). The results in the current study demonstrated that among the components of PSQI, compared to the attack-free period, scores except the one assessing the need for medication to sleep showed a statistically significant increase. No significant correlations were found between BaSIQS and PSQI scores with age, number of attacks, duration of icatibant effect, annual hospital visits, AECT, or AAS in our cohort. These findings suggest that targeted interventions to prevent and manage attack-related symptoms, particularly pain, could benefit sleep quality.

To our knowledge, no study has assessed sleep disorders in HAE patients. However, research involving patients with other inborn errors of immunity noted that nearly half had a total PSQI score greater than 5 (5). Similarly, our study findings showed that half of HAE patients had a median PSQI score of 5 in the attack-free period. The significant deterioration in sleep quality during HAE attacks, as evidenced by higher PSQI and BaSIQS scores, highlights the profound impact of acute episodes on patients’ overall well-being.

Whereas a specific study on patients receiving androgens for long-term HAE prophylaxis found nearly half reported persisting symptoms of agitation, sleeplessness, or insomnia (45.6%) (27, 31). In the current study, since only two patients were treated with androgens as LTP, data is limited to compare to the previous report. As a suggestion, it can be stated that to improve disease control and sleep quality in line with QoL, targeted therapies for LTP of HAE should be widely available rather than nonspecific medications such as androgens or tranexamic acid.

This study has several limitations, including a focus on insomnia symptoms and sleep quality without addressing other sleep disorders, a small sample size typical for rare diseases like HAE, and potential biases from subjective questionnaire responses. Study design primarily relies on self-reported questionnaire data, which, while valuable for assessing subjective sleep experiences, may be influenced by recall bias and individual perception. Future research integrating objective sleep assessments would provide a more comprehensive evaluation and strengthen the reliability of the results. Secondly, concomitant depression and anxiety were not assessed in our patients since many studies already showed the increased prevalence of anxiety and depression in HAE patients. The small sample size (*n* = 22) and the single-center design may limit the generalizability of our findings. Therefore, larger multicenter studies are needed to validate these results and enhance statistical power. Despite these, the current study adds to the growing body of evidence on sleep disturbances in HAE patients by evaluating all domains of PSQI and BaSIQS, underscoring the need for comprehensive sleep assessments in this population. Using validated questionnaires provides a practical approach to assessing sleep quality in HAE patients and can be easily integrated into routine clinical care.

In conclusion, this study indicates that most HAE patients experience poor sleep quality and disturbances, even during attack-free periods. Future studies should investigate the utility of objective measures, such as polysomnography, to further clarify the nature and extent of sleep disturbances in this rare group. Patients should be evaluated for sleep disorders during clinical assessments and referred for diagnosis as needed. Prophylactic therapies and psychological interventions regarding sleep outcomes could offer valuable insights into optimizing patient care. Ultimately, this approach may improve health-related quality of life in accordance with disease control.

## Data Availability

The original contributions presented in the study are included in the article/Supplementary material, further inquiries can be directed to the corresponding author.
