# Supplementary material for: Subjective assessment of sleep quality in adult patients with hereditary angioedema
Source: Front Neurol. 2025 Apr 16;16:1555562. doi: 10.3389/fneur.2025.1555562 (PMC12042759; doi:10.3389/fneur.2025.1555562)
Supplement: Supplementary file 1 [file Table_1.docx]

| Patient (P) | C4  (0.1-0.4 g/L) | C1 inhibitor antigen  (21-38 g/L) | C1 inhibitor function (70-132 %) | Diagnosis of HAE |
| --- | --- | --- | --- | --- |
| P1 | 0.03 | <8 | 17 | Type I |
| P2 | 0.03 | <8 | 8.2 | Type I |
| P3 | <0.02 | <8 | 22.9 | Type I |
| P4^*^ | 0.2 | 14.8 | 46.6 | Type I |
| P5 | 0.05 | 7.8 | 8.2 | Type I |
| P6 | 0.03 | 9.2 | 12.7 | Type I |
| P7 | <0.02 | 7.1 | 11.3 | Type I |
| P8 | 0.08 | 30 | 21.3 | Type II |
| P9 | <0.02 | <8 | 8.2 | Type I |
| P10 | <0.08 | 10.4 | 12.3 | Type I |
| P11 | <0.08 | 9.2 | 8.2 | Type I |
| P12 | <0.02 | <8 | 16.8 | Type I |
| P13^*^ | 0.1 | 45 | 19.3 | Type II |
| P14 | <0.02 | 9.9 | 14.4 | Type I |
| P15^⁑^ | 0.24 | 27.3 | 115 | Type III |
| P16 | <0.02 | 8 | 29.1 | Type I |
| P17 | <0.08 | 8 | 19.8 | Type I |
| P18 | <0.08 | 8 | 8.2 | Type I |
| P19 | <0.08 | <8 | 8.2 | Type I |
| P20 | 0.05 | 4.4 | 3.4 | Type I |
| P21 | <0.08 | <8 | 23 | Type I |
| P22 | <0.08 | <8 | 21 | Type I |

**Table S1.**The results of patients with Hereditary Angioedema

*Two patients receiving Danazole treatment, ⁑patient with normal C1 inhibitor
